# Feeling the pain of others is associated with self-other confusion and prior pain experience

**DOI:** 10.3389/fnhum.2013.00470

**Published:** 2013-08-14

**Authors:** Stuart W. G. Derbyshire, Jody Osborn, Steven Brown

**Affiliations:** ^1^Department of Psychology and A^*^STAR-NUS Clinical Imaging Research Centre National University of SingaporeSingapore; ^2^Psychology Department, Newman University CollegeBirmingham, UK; ^3^Rowett Institute of Nutrition and Health, University of AberdeenAberdeen, UK

**Keywords:** pain, empathy, illusion, vicarious, sensitivity

## Abstract

Some chronic pain patients and healthy individuals experience pain when observing injury or others in pain. To further understand shared pain, we investigated perspective taking, bodily ownership and tooth pain sensitivity. First, participants who reported shared pain (responders) and those who did not (non-responders) viewed an avatar on a screen. Intermittently, 0–3 circles appeared. Sometimes the participant's and avatar's perspective were consistent, both directly viewed the same circles, and sometimes inconsistent, both directly viewed different circles. Responders were faster than non-responders to identify the number of circles when adopting a consistent perspective. Second, participants sat with their left hand hidden while viewing a rubber hand. All participants reported an illusory sensation of feeling stroking in the rubber hand and a sense of ownership of the rubber hand during synchronous stroking of the rubber and hidden hand. The responders also reported feeling the stroking and a sense of ownership of the rubber hand during asynchronous stroking. For experiment three, participants with either low, moderate, or high tooth sensitivity observed a series of images depicting someone eating an ice-popsicle. Low sensitivity participants never reported pain. In contrast, moderate and high sensitivity participants reported pain in response to an image depicting someone eating an ice popsicle (4 and 19% of the time, respectively) and depicting someone eating an ice-popsicle and expressing pain (23 and 40%, respectively). In summary, responders have reduced ability to distinguish their own and others' visual perspective and enhanced ability to integrate a foreign arm into their bodily representation. The tendency to share pain is also enhanced when an observed pain is commonly experienced by the observer. Shared pain may therefore involve reactivation of pain memories or pain schema that are readily integrated into a self perspective and bodily representation.

## Introduction

A significant number of patients with phantom limb pain report pain in response to the observation of injuries or other thoughts and images associated with pain (Giummarra and Bradshaw, 2008; Fitzgibbon et al., 2009, 2010; Giummarra and Moseley, 2011) and some patients report feeling touch when observing others being touched (Goller et al., 2013). Normal control populations also report feeling pain when observing images or videos of others' injuries (Osborn and Derbyshire, 2010) and some normal subjects also report feeling touch sensations when observing another person being touched (Banissy et al., 2009). Thus, there is evident capacity for shared sensory experience, including physically painful experience, that extends beyond a shared emotional empathic response (Singer et al., 2004; Botvinick et al., 2005; Jackson et al., 2005, 2006) or a metaphorical shared pain experience (Eisenberger et al., 2003; MacDonald and Leary, 2005). The mechanisms behind such shared physical experiences remain uncertain and here we investigate the influence of visual perspective taking, bodily ownership, and prior pain experience.

Visual perspective taking refers to the ability to predict what another person sees (Michelon and Zacks, 2006). Increased ability to process information in the first person perspective relative to the third person perspective suggests that visual perspective may play a crucial role in the representations of self and the representations of other (Jeannerod and Anquetil, 2008). Successful social interaction requires inferring the visual and mental perspectives of others. The ability to infer what another person can see implies disengaging from the self visual perspective and adopting the visual perspective of another (Samson et al., 2010). Self perspective is considered as a default egocentric bias that is corrected or inhibited when trying to understand others (Keysar and Henly, 2002). Studies have shown that some participants can suppress self perspective more quickly, suggesting that some individuals more readily adopt the perspective of others (Epley et al., 2004; Samson et al., 2005). Here it is hypothesized that individuals who report feeling pain in response to seeing others' injuries, known as pain responders, will have fewer processing constraints from a first to third person perspective and thus will map across visual perspectives more quickly and easily relative to non-pain responders who never report feeling pain in response to seeing others' injuries.

A way of exploring bodily ownership is to utilize the rubber hand illusion (Botvinick and Cohen, 1998). The rubber hand illusion is induced when a participant sits with their hand and arm hidden by a partition while viewing a rubber hand and arm in an anatomically appropriate position such that their hand and arm could be in the position of the rubber hand and arm. The experimenter then synchronously strokes both the hidden hand and the rubber hand. Within a few minutes, most subjects report that the stroking sensation no longer feels as if it is coming from their hidden real hand but is actually emanating from the observed rubber hand. This illusory sensation and feeling of ownership over the rubber hand is thought to come about through multisensory integration of visual, tactile, and proprioceptive information (Haggard and Tsakiris, 2005). After establishing the illusion, “injuring” the rubber hand by bending back a finger causes an elevated skin-conductance response (Armel and Ramachandran, 2003), although skin-conductance is a general measure of arousal and so may not be linked to a feeling of threat or pain. Threatening the rubber hand with a knife, however, activates regions of the brain associated with anticipated pain (Ehrsson et al., 2007). A noxious stimulus can also result in pain mislocated into the rubber hand (Capelari et al., 2009; Mohan et al., 2012). Pain evoked by someone else's injury seems to involve a misattribution of threat from the location of the observed injury to the same location on the observer (Osborn and Derbyshire, 2010). Thus, it is hypothesized that pain responders will have stronger illusory sensation and feeling of ownership over the rubber hand during the rubber arm illusion compared with non-responders.

The role of prior pain experience when sharing pain through observation has been explored in several reports on phantom limb pain (Fitzgibbon et al., 2009, 2010). Phantom limb patients have reported experiencing heightened phantom pain when observing, thinking about, or inferring the pain of another. At least sometimes the pain is linked to the patient's particular history. For example, one patient experienced pain in his lower limb stumps when observing someone walking barefoot (Fitzgibbon et al., 2009). Following a particularly distressing and painful emergency caesarean section, another patient reported shooting pains from the groin that radiated down the legs when hearing about others' trauma (Giummarra and Bradshaw, 2008). These case studies imply that shared pain experience might reactivate prior or ongoing pain sensations. Here it is hypothesized that participants with high tooth sensitivity will be more likely to report a shared pain experience when viewing someone expressing pain while consuming an ice popsicle than participants without tooth sensitivity. Tooth sensitivity is a common dental problem characterized by short, sharp pain from the teeth in response to a variety of stimuli often including cold stimuli (Addy, 1992). Thus, tooth sensitive participants were considered a convenient population to test the possibility that shared pain experience can involve reactivation of previous pain.

The three studies described here will provide insight into mechanisms of shared pain experience. Specifically, it is possible, but yet to be demonstrated, that shared pain involves readily taking the perspective of another person, which may be indexed by more rapid orientation to the visual perspective of others (experiment one); readily mapping the location of injury of another to the self, which may be indexed by stronger sense of mislocating sensation into a rubber arm (experiment two); and readily integrating the observed pain of another into a personal historical schema, which may be indexed by activation of tooth pain in those with and without tooth sensitivity (experiment three).

## Materials and methods

### Experiment one

Twenty six self selecting participants (3 males; mean age = 19; range = 18–21) provided informed consent and took part in experiment one for course credit. All participants were examined in a single session by a female experimenter. Participants observed a series of images or videos depicting injury and rated any pain responses (Osborn and Derbyshire, 2010). If a participant reported pain they were asked additional questions to explore the nature of the pain experience and to ensure that feelings of unpleasantness or visceral reaction were clearly discriminated from somatic signs of noxious experience. Further questions included: “How long did the pain sensation last?,” “How would you describe the pain sensation you felt?,” “How did it feel?,” “Have you previously experienced a similar kind of pain following an injury or other problem?,” and “Do you get this type of pain in everyday life or when you watch a movie?” The investigator asked additional questions to clarify the nature of the experience as somatic, rather than just visceral or emotional, when necessary. Those responding to at least one image or video with a pain response that was not just an emotional or “gut” reaction were assigned as a responder to yield ten responders and sixteen non-responders.

**Figure 1:**
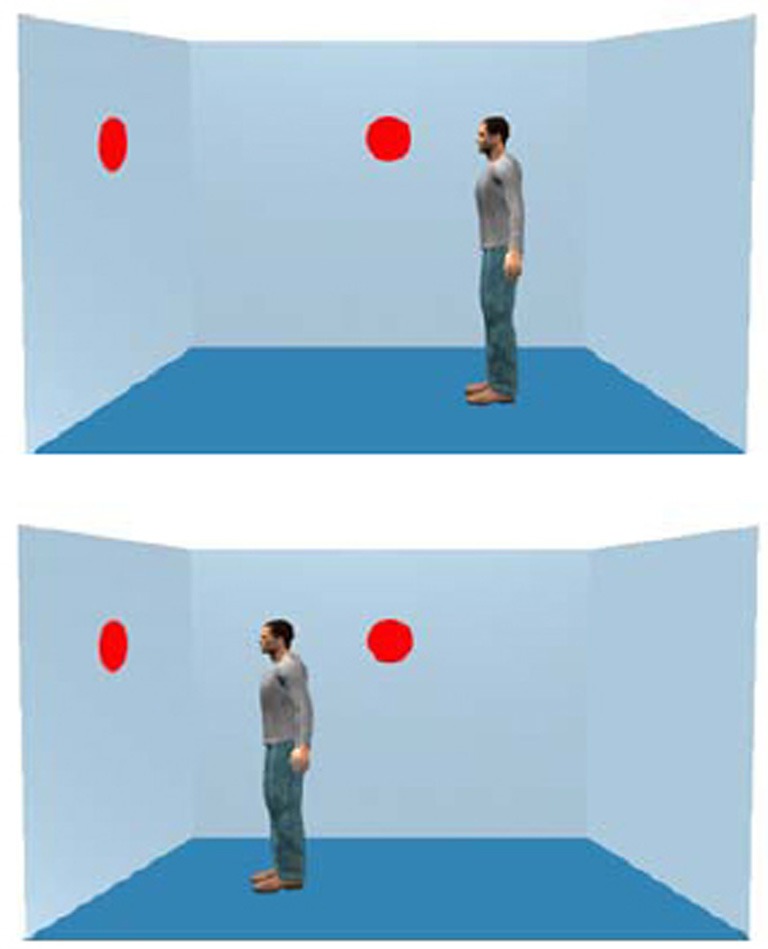
**The figure shows two dots and an avatar observing two dots (top), consistent with the participant's viewpoint or observing one dot (bottom), inconsistent with the participant's viewpoint**.

All participants then took part in a reaction time experiment involving an avatar viewed on a computer screen surrounded by three virtual walls (following the design of Samson et al., 2010). A female avatar was used for female participants and a male avatar for male participants. At intermittent intervals, 0–3 circles were presented either on the wall facing the avatar or on the wall facing away from the avatar (Figure 1).

The participant could always see the number of circles. In half of the trials the avatar was observing the same number of circles as the participant such that the avatar's and the participant's perspective were consistent. In the other half of the trials the avatar observed a different number of circles to the participant such that the avatar's and the participant's perspective were inconsistent. The position of the avatar was randomized for each trial. Prior to seeing the room, participants were cued to adopt either their own perspective, which was written as “you,” or the perspective of the avatar, which was written as “he” or “she” as appropriate. For half of the trials the participants adopted the perspective of the avatar and for the other half they adopted their own perspective. After viewing the screen for 750 ms, participants were asked to identify the number of circles on the wall from their adopted perspective (self or other) as quickly as possible. There were 96 trials in total. Time taken to press the button was automatically recorded. Reaction times 2.5 standard deviations outside the mean were removed as outliers.

### Experiment two

Fifty two new self selecting participants (all females; mean age = 20; range 18–22) provided informed consent and took part in experiment two for course credit. All participants observed the images or videos depicting injury, as before, and 19 reported pain to at least one image or video (responders).

All participants then took part in a test of the rubber hand illusion. A purpose built partition and cover allowed each participant to sit with their left arm and hand hidden from view. All participants wore a yellow rubber glove on their right hand and were seated with their arms resting on a table in front of them. The partition obscured their view of their left arm and hand and the gloved rubber arm and hand was placed on the visible side of the partition positioned where the participant indicated it felt natural, “as though my own left arm could comfortably be resting there.” Two experimental conditions, synchronous stroking of the participant's left hand and the rubber hand and asynchronous stroking of the participant's hand and the rubber hand then followed and continued for 1 min. The order of conditions (synchronized or asynchronized stroking) was randomized across participants. Immediately after finishing each condition, the participant was asked to fill out the Botvinick and Cohen (1998) questionnaire. The Botvinick and Cohen (1998) questionnaire includes eight items describing perceptual qualities associated with the rubber arm illusion. The first three items have been previously demonstrated as highly correlated with the rubber hand illusion (Botvinick and Cohen, 1998). Participants were asked to what extent they agreed or disagreed with each statement from 3 (strong agreement that the sensation or experience was felt) to −3 (strong disagreement).

### Experiment three

Sixty-three new participants (7 males; mean age 20; range 18–21) were recruited by advertisement from the University of Birmingham and surrounding area. All participants provided consent. Participants completed a “teeth sensitivity” questionnaire which included the following items: “how much pain do you feel when you eat cold foods (0 = none, 10 = most pain imaginable),” “how sensitive do you think your teeth are (0 = not at all, 10 = extremely),” and “do you receive treatment for sensitive teeth (Y/N).” Participants who scored 15 or above and who reported receiving treatment for teeth sensitivity were categorized as high sensitivity (*n* = 20). Participants who scored 10 or below and who reported not receiving treatment for sensitive teeth were categorized as low sensitivity (*n* = 21). The remaining participants were categorized as moderate sensitivity (*n* = 22).

**Figure 2:**
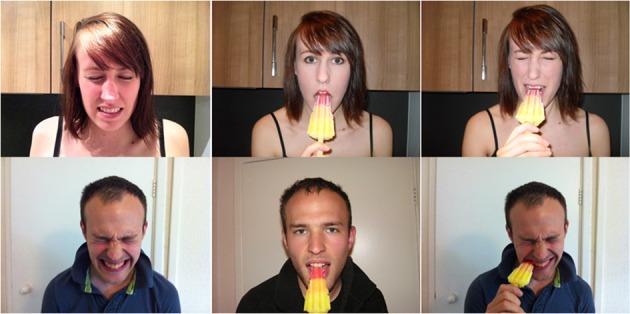
**The figure shows the images used for experiment three. On the left is pain alone, in the middle is the ice popsicle alone and on the right is pain with the ice popsicle**.

Participants viewed a series of six images of a male or female face depicting three conditions: expressing pain, eating an ice-popsicle and not expressing pain and eating an ice-popsicle and expressing pain (see Figure 2). The final image was expected to elicit a greater frequency of pain in participants with tooth sensitivity. The image expressing pain alone was intended to control for evoked pain independent of tooth sensitivity similar to previous studies (Osborn and Derbyshire, 2010) and the image not expressing pain and eating an ice-popsicle controlled for the influence of observing an act that could cause the observer pain. The images were presented for three seconds and then the participants were asked if they felt any sensation of pain while viewing the image. It was emphasized that the pain should be felt in the body and general feelings of unpleasantness or unease should not be recorded as painful (following Osborn and Derbyshire, 2010). Participants who reported pain also rated the intensity of their pain using a visual analogue scale (VAS) (anchored at 0 for no pain and at 10 for most pain imaginable) and the short-form McGill Pain Questionnaire (MPQ). All participants completed the Interpersonal Reactivity Index (IRI) to assess trait empathy and rated their empathic feelings (state empathy) toward the person in each image using a numerical rating scale from zero (indicating no compassion, warmth, or sympathy toward the depicted person) to 10 (indicating the most compassion, warmth, or sympathy imaginable).

## Results

### Experiment one

**Figure 3:**
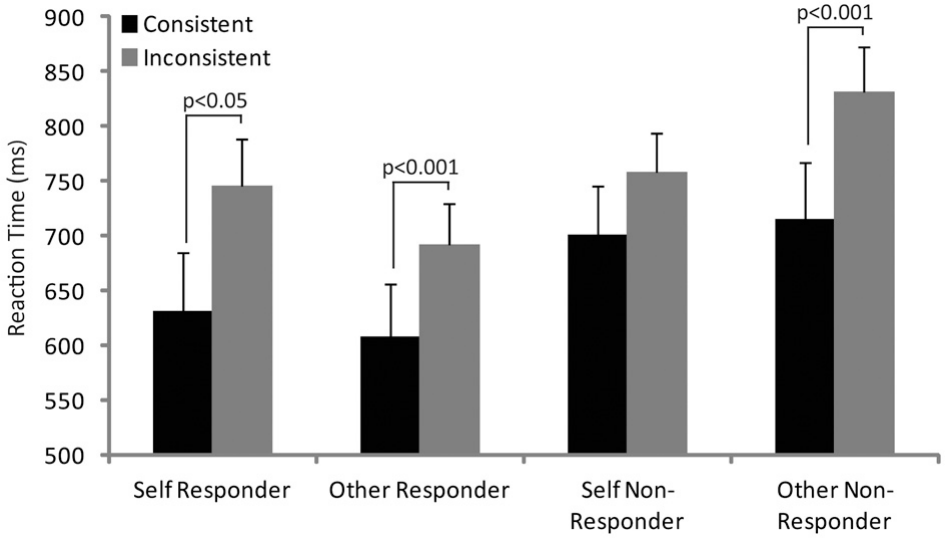
**The figure shows the reaction times in responders and non-responders during consistent and inconsistent trials when adopting self and other perspective**. *Post-hoc* significant differences are indicated.

Figure 3 shows the reaction times for the consistent and inconsistent trials, when adopting a self or other perspective, for the responder and non-responders separately. Participants were faster across groups and conditions for the consistent trials. The difference between consistent and inconsistent trials when adopting a self perspective, however, was greater for the responders compared to the non-responders. In contrast, the difference when adopting an other perspective was greater for the non-responders compared with the responders. Prior to analysis, the data were examined for violations of normality including skewness and violations were not exceptional (measures of skewness ranged from 0.1 to 1.0). The data were also tested for equality of variance and no violation of unequal variance was evident (*p* = 0.48). Thus, a 2 (consistent/inconsistent) × 2 (self/other perspective) × 2 (responder/non-responder) ANOVA was used to formally assess the data. The ANOVA confirmed a main effect of consistency [*F*_(1, 24)_ = 28.6, *p* < 0.001] a consistency by perspective interaction [*F*_(1, 24)_ = 12.6, *p* < 0.01] and a trend toward a three way interaction of consistency, perspective and group [*F*_(1, 24)_ = 3.7, *p* = 0.07]. No other effects reached or trended toward significance.

*Post-hoc* paired *t*-tests were used to explore the interaction of consistency with perspective and revealed significant differences between consistent and inconsistent trials when adopting the self (*t* = 2.9, *p* < 0.05) and other (*t* = 6.4, *p* < 0.001) perspective in responders but only when adopting the other perspective in non-responders (*t* = 5.4, *p* < 0.001). No other differences reached significance.

### Experiment two

Prior to analysis, the Botvinick and Cohen questionnaire data were examined for violations of normality including skewness and violations were not exceptional (measures of skewness ranged from −0.75 to 0.04). The data were also tested for equality of variance and a violation of unequal variance was evident (*p* < 0.001) and so corrected degrees of freedom were implemented. The data were first examined with a 2 (synchronous/asynchronous stroking) × 8 (question) × 2 (responder/non-responder) ANOVA for formal assessment. The results revealed significant heterogeneity across questions [*F*_(4.7, 230)_ = 22.7, *p* < 0.001] as well as a significant main effect of synchronicity [*F*_(1, 49)_ = 5.4, *p* < 0.05]. Question one (“It seemed as if I were feeling the touch of the paintbrush in the location where I saw the rubber hand touched”) received the highest score and question eight (“It felt as if my real hand were turning rubbery”) the lowest score. Scores were higher during synchronized compared with asynchronized stroking. The interactions of question with group and question with condition were significant [*F*_(4.7, 230)_ = 2.3, *p* < 0.05; *F*_(4.9, 242)_ = 2.4, *p* < 0.05] but there was no significant three-way interaction of question, condition and group [*F*_(4.9, 242)_ = 1.8, *p* = 0.11]. No other effects reached, or approached, significance.

**Figure 4:**
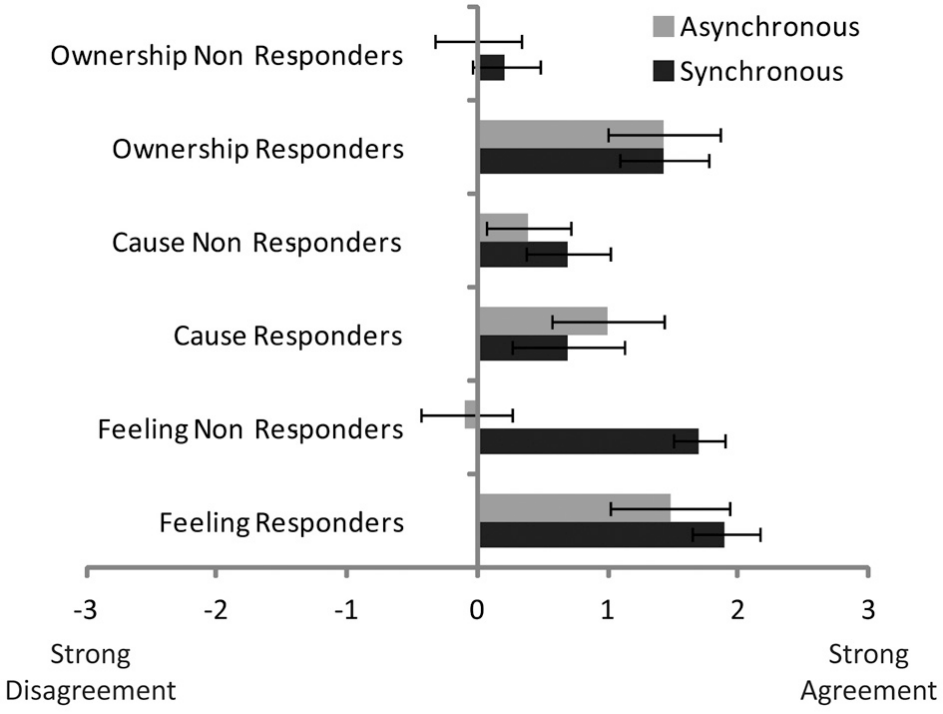
**The figure shows the group mean ratings for feeling the touch of the brush on the rubber hand (“Feeling”), reporting the touch to be caused by the brush (“Cause”) and feeling as if the rubber hand was the participant's hand (“Ownership”)**.

The data were explored further by analyzing the three critical questions relating to feeling the stroking of the brush, feeling the stroking being caused by the touch of the brush on the rubber hand, and feeling ownership of the rubber hand, using a 2 (synchronous/asynchronous stroking) × 3 (question) × 2 (responder/non-responder) ANOVA. The results are illustrated in Figure 4.

From Figure 4 it can be seen that the responders tend to have greater responses than non-responders, largely because the responder scores remained high even during asynchronous stroking. Formal analysis confirmed the main effect of group [*F*_(1, 50)_ = 5.8, *p* < 0.05], synchronicity [*F*_(1, 50)_ = 4.3, *p* < 0.05] and question [*F*_(2, 100)_ = 3.6, *p* < 0.05] as well as a significant interaction of synchronicity with group [*F*_(1, 50)_ = 3.6, *p* < 0.05] and synchronicity with question [*F*_(2, 100)_ = 9.7, *p* < 0.001] but no three way interaction of synchronicity, question, and group [*F*_(2, 100)_ = 2.1, *p* = 0.11]. No other effects reached, or came close, to significance.

### Experiment three

High sensitivity participants reported more pain in the presence of the ice-popsicle than the other two groups. The low sensitivity participants never reported pain (see Table 1). A series of χ^2^ analyses revealed a significant effect of group for pain responses to the pain without ice popsicle picture [χ^2^_(2)_ = 7.4, *p* < 0.05], the no pain with ice popsicle picture [χ^2^_(2)_ = 19.1, *p* < 0.001] and the pain with ice popsicle picture [χ^2^_(2)_ = 30.2, *p* < 0.001].

**Table 1:** **Shows the number of times participants in the high, moderate (Mod) and low sensitivity groups responded with or without pain for the three image conditions**.

	**Pain without ice-popsicle**			**No pain with ice-popsicle**			**Pain with ice-popsicle**		
	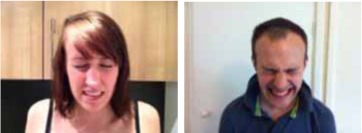			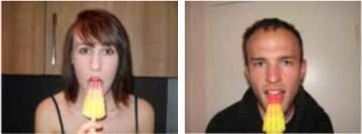			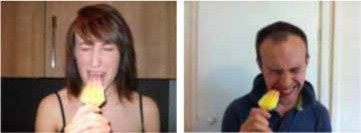		
	**High**	**Mod**	**Low**	**High**	**Mod**	**Low**	**High**	**Mod**	**Low**
Pain	4	9	0	15	4	0	32	20	0
No pain	76	79	64	65	84	64	48	68	64

**Figure 5:**
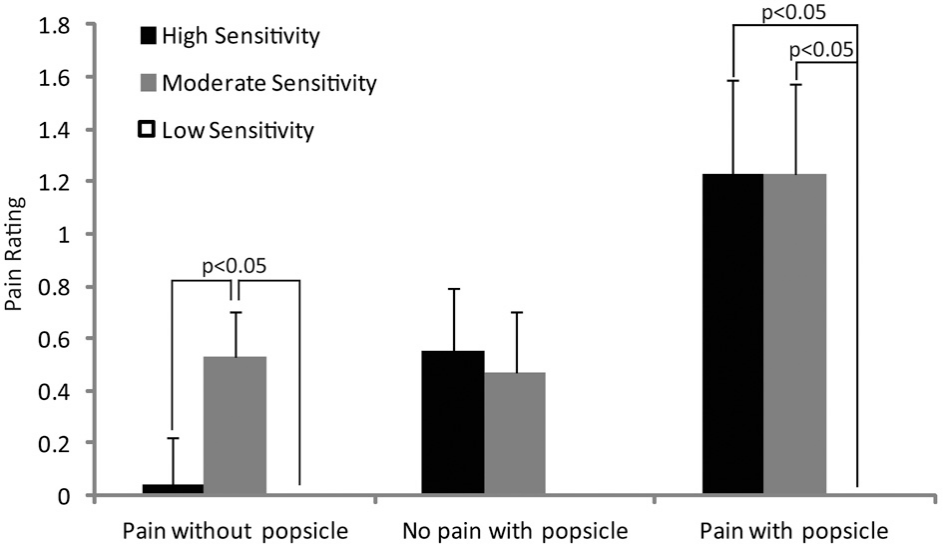
**The figure shows the mean VAS pain ratings for each tooth sensitivity group in response to each image type**. *Post-hoc* significant differences are indicated.

The mean VAS pain ratings for each group in response to each class of image are shown in Figure 5. The data were analyzed using a 3 (group—high, moderate or low sensitivity) × 3 (image—pain without ice popsicle, no pain with ice popsicle or pain without ice popsicle) × 2 (gender—male or female picture) ANOVA. The main effects of group and image were significant [*F*_(2, 60)_ = 3.7, *p* < 0.05; *F*_(2, 120)_ = 9.1, *p* < 0.001] and so was the interaction of image with group [*F*_(4, 120)_ = 2.6, *p* < 0.05]. *Post-hoc* pairwise comparisons revealed significantly (*p* < 0.05) greater pain in the moderate sensitivity group compared with the high and low sensitivity groups for the pain without ice popsicle picture. Both the high and moderate sensitivity groups reported significantly greater pain compared with the low sensitivity group for the pain with ice popsicle picture. No other differences reached significance.

**Figure 6:**
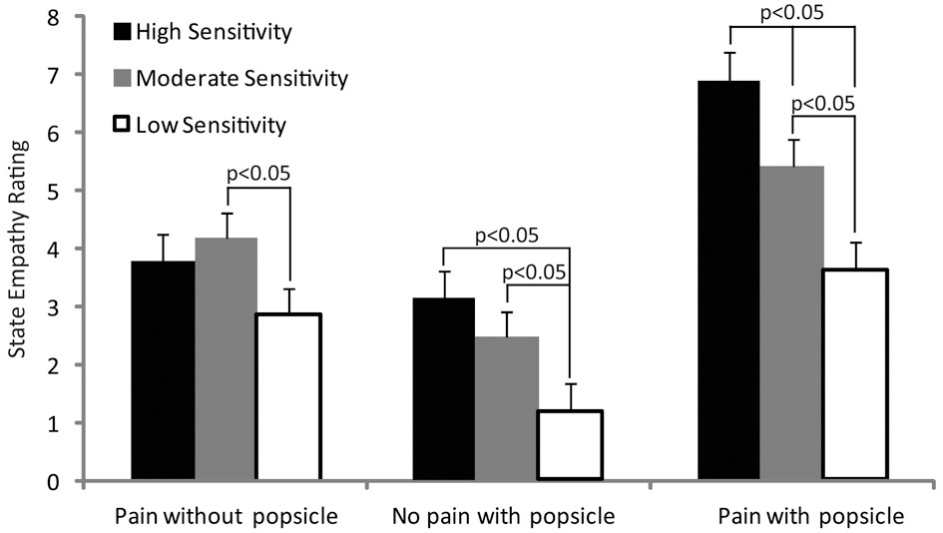
**The figure shows the mean state empathy ratings for each tooth sensitivity group in response to each image**. *Post-hoc* significant differences are indicated.

Out of the 79 pain reports, 74 were reported in the teeth, face, or head (two were reported in the lower back, two in the right foot and one in the chest). The pain was typically described as sharp (used 57 times), shooting (39), aching (36), and throbbing (30). Trait empathy was similar across groups (high = 78, moderate = 79, low = 83) but state empathy differed according to group as shown in Figure 6. State empathy data were formally analyzed using a 3 (group—high, moderate or low sensitivity) × 3 (image—pain without ice popsicle, no pain with ice popsicle or pain without ice popsicle) × 2 (gender—male or female picture) ANOVA. The main effects of group and image were significant [*F*_(2, 60)_ = 7.2, *p* < 0.01; *F*_(2, 120)_ = 67.7, *p* < 0.000] and so was the interaction of image with group [*F*_(4, 120)_ = 3.8, *p* < 0.01]. *Post-hoc* pairwise comparisons revealed significantly (*p* < 0.05) higher ratings in the moderate sensitivity compared with low sensitivity group for the pain without ice popsicle picture; higher ratings in the high sensitivity group compared with moderate and low sensitivity groups and higher ratings in the moderate sensitivity compared with the low sensitivity group for the pain with ice popsicle picture; and higher ratings in the high and moderate sensitivity groups compared with the low sensitivity group for the no pain with ice popsicle picture. No other differences reached significance.

## Discussion

Three experiments, involving different samples of participants who do (responders) and do not (non-responders) report directly sharing others' pain, were conducted to further understand the mechanisms of shared pain experience. Experiment one provides evidence that all participants suffer interference from someone else's visual perspective even when explicitly instructed to adopt their own perspective, which replicates previous findings (Samson et al., 2010). Experiment one, however, also provided evidence for greater interference effects when adopting the perspective of the other compared with the self and this was driven in part by reduced interference when adopting a self perspective. This reduced interference was especially noticeable for the non-responders although the critical three-way interaction only trended toward significance. Experiment two provides evidence for a stronger integration of the rubber arm into bodily representation for responders compared with non-responders. Experiment three provides evidence that being a pain responder is increased if the observed pain is congruent with a current pain sensitivity. Specifically, people with high and moderate tooth sensitivity were significantly more likely to report pain when observing an image of someone biting into an ice-popsicle, especially if the person expressed pain while biting. In combination, these findings suggest that responders can more readily adopt the perspective of others, more readily integrate foreign body parts into their own body schema and more readily experience pain when observing a behavior that has caused them pain in the past.

The visual perspective taking task used here required the participants to make inferences about what another can or cannot see (Newcombe, 1989). Correctly inferring what another can see requires the viewer to inhibit their egocentric viewpoint and adopt the other's visual perspective. This inhibition of egocentric or self viewpoint can also contribute to understanding the thoughts and feelings of others by reducing the influence of the predominant, egocentric, self perspective (Vogeley et al., 2001). Inhibiting the self-perspective and adopting another's mental perspective is considered an essential part of empathic understanding (Davis, 1980). Imagining a “self” perspective while viewing someone in pain, for example, may aid confusion between self and other perspectives (Lamm et al., 2007).

Visual perspective taking does not necessarily require any inference regarding the mental state of the other (Newcombe, 1989; Aichhorn et al., 2006). Inferring the mental state of another and then sharing that state, as is the case with empathy, may involve subjectively adopting the cognitive perspective of the other to understand what he or she is thinking. There is a distinction between the ability to shift visual perspective, which is a low level skill, and the ability to empathize by thinking what someone else is thinking or feeling what someone else is feeling. Presumably lower level skills, including automatic visual perspective taking, contribute to higher level skills, including empathy (Samson et al., 2010). It is possible that the low level mechanism of visual perspective taking contributes to the emotional experience of empathy for another in pain, which correlates with vicarious sensation of both touch and pain (Singer et al., 2004; Banissy and Ward, 2007), but that at least some components of empathy remain independent of vicarious sensation.

Pain responders reported similar experiences of the rubber hand illusion in the asynchronous stroking as the synchronous stroking condition. Previous research has demonstrated an attenuation of the illusion during asynchronous stroking, stronger than observed here in the responders (Botvinick and Cohen, 1998; Ehrsson et al., 2005; Tsakiris and Haggard, 2005; Tsakiris et al., 2006; Makin et al., 2008; Moseley et al., 2008; Aspell et al., 2009). Although aspects of the rubber hand illusion can be generated with asynchronous stroking, synchronous stroking is considered as particularly important for generating feelings of ownership over an external body part (Makin et al., 2008; Tsakiris, 2010). Here, reports of body ownership during asynchronous stroking suggest that strong correlations between tactile and visual input are less important for ownership over another person's hand for pain responders.

It is possible that visual information dominates tactile information in driving feelings of ownership for responders. Previous research has demonstrated that viewing a body other than one's own tends to activate a visual simulation mechanism that rapidly readies the somatosensory system to experience observed physical events (Longo et al., 2011; Cardini et al., 2011, 2012, 2013). Pain responders might be at the extreme end of this tendency, partly explaining their experience of pain in response to someone else's injury but possibly also explaining why responders were equally affected by congruous as by incongruous stroking. It is possible, at least for responders that simply viewing the rubber hand in an anatomically appropriate position resulted in rapid somatotopic integration sufficient to compensate for the incongruent tactile stimulation that followed. Some participants did spontaneously report feeling the illusion as soon as they placed their arms into the apparatus but this spontaneous report was not systematically investigated. Future studies might address whether the illusion is spontaneously generated more easily in responders compared to non-responders. A more flexible sense of body part ownership may partially explain how responders relocate an observed injured body part of another to themselves, producing pain in the self.

Participants who reported sensitivity to pain when eating cold foods were significantly more likely to report pain sensation after observing others eat cold foods. This finding supports the idea that we feel the pain of others more if we have experienced the pain ourselves and implies a merging of self and other. Shared pain experience was also associated with increased state empathy but not trait empathy. While it is generally accepted that representations of self and other overlap during the experience of empathy, it is less clear how self/other merging occurs. We may feel what it is like for someone else to be in pain (*Like them*) or we may feel what it is like for us to be in pain (*Like us*) (Decety and Sommerville, 2003). *Like them* depends less on self representations of pain and more on “other” oriented empathic processes. *Like us* depends more on “self” oriented representations of pain and may plausibly be less dependent on “other” oriented empathic processes. Here participants with self experience of pain from cold food had increased pain experience when observing someone eat an ice-popsicle. Thus, our findings point more toward *Like us* mechanisms than toward *Like them*. *Like them* would have been expected to reveal no pain when observing someone biting an ice-popsicle but not feeling pain and equivalent pain when observing a facial expression of pain with and without the ice-popsicle.

Interestingly, participants who reported sensitivity to pain responded with increased pain intensity both to the pain with ice-popsicle picture and to the no pain with ice-popsicle picture. Previous research has used images depicting injuries that would hurt the observer if the same thing happened to them but are also clearly likely to hurt the person depicted (Morrison et al., 2004; Singer et al., 2004; Jackson et al., 2005; Osborn and Derbyshire, 2010). Here it is demonstrated that images depicting events that would only hurt the observer (if they have sensitive teeth) can cause pain in the observer. In this instance, respondents are not responding to the pain of the other but are responding to the fact that the action depicted, biting into an ice-popsicle, could cause them pain. At the same time, participants without tooth sensitivity, but with similar high levels of trait empathy to those with tooth sensitivity, did not respond with pain to the images depicting someone expressing pain while biting into an ice-popsicle. These findings provide a double dissociation away from an explanation of vicarious pain based on empathy with some participants responding despite the image not depicting pain, and thus reducing or eliminating a pain induced empathic response, and some participants not responding despite a pain induced empathic response to the pain images.

It is also interesting that there was more pain reported by the moderate sensitivity group to the pain without ice-popsicle image. This finding further suggests that the pain of the high sensitivity group is driven largely by the depiction of something that could hurt them rather than being a general response to an expression of pain. It remains uncertain, however, why the moderate pain group reported more pain than both the low and high sensitivity groups.

Including an additional control picture only depicting an ice-popsicle would have established if merely observing a salient affective stimulus causes pain in participants with sensitive teeth. Including this control image was rejected because an ice-popsicle alone was thought to be unlikely to generate pain. By itself, an ice-popsicle cannot induce pain, and so the participants would have no history of pain from ice-popsicles *per se*, only from biting into them. In addition, the possibility of causing a diminished response from showing many ice-popsicle pictures was also considered. Nevertheless, this lack of control limits the interpretation.

A number of additional limitations also mean that the results reported here should be treated with caution before replication. In particular, the critical interaction effect for experiment one only trended toward significance and many of our response measures relied on subjective assessment. Similarly for experiment two, there was insufficient statistical support for a significant three-way interaction that might indicate more specific influences of responder vs. non-responder during the rubber hand illusion. All studies were performed using convenience samples with numbers comparable to previous research. It is possible that the studies were simply underpowered to reveal smaller effects. All experiments involved a relatively limited demographic (mostly young females) that may introduce bias and difficulties in generalizing the findings. Experiment three, in particular, may involve demand characteristics driving pain report in those with sensitive teeth when viewing the ice-popsicle images. Future studies may benefit from including objective measures, such as GSR, alongside subjective report, to address at least some of these potential biases. We are currently investigating brain activation using fMRI with responders and non-responders to the ice popsicle.

## Conclusions

The studies reported here demonstrate that responders more readily adopt the perspective of an other and more readily integrate a foreign body part into the self. The number of responders also increases when the observed pain is one that the participant is familiar with from their own history. Thus, experiencing pain when observing the pain of someone else may rely upon the integration of the other into a self orientated representation of injury or pain.

### Conflict of interest statement

The authors declare that the research was conducted in the absence of any commercial or financial relationships that could be construed as a potential conflict of interest.
